# Determination of optimal implantation site in central venous system for wireless hemodynamic monitoring

**DOI:** 10.1016/j.ijcha.2020.100510

**Published:** 2020-04-03

**Authors:** Tejaswini Manavi, Patricia Vazquez, Muhammad Tubassam, Junaid Zafar, Faisal Sharif, Haroon Zafar

**Affiliations:** a Cardiovascular Research & Innovation Centre, National University of Ireland Galway, Ireland; b Lambe Institute for Translational Research, School of Medicine, National University of Ireland Galway, Ireland; c Department of Vascular Surgery, University Hospital Galway, Ireland; d Faculty of Engineering, Government College University Lahore, Pakistan; e CÚRAM-SFI Centre for Research in Medical Devices, Galway, Ireland; f Department of Cardiology, University Hospital Galway, Ireland; g BioInnovate, Ireland

**Keywords:** Hemodynamic sensor, Heart failure, Pulmonary artery, Central venous system

## Abstract

**Background/purpose:**

In recent years, treatment of heart failure patients has proved to benefit from implantation of pressure sensors in the pulmonary artery. Despite this, pulmonary artery pressure is related to the left ventricle, and cannot provide information on the right side of the heart. By contrast, pressure in the central venous system is directly connected to the right atrium and could potentially predict a wider range of heart failure conditions. The purpose of this work is to find an optimal site for implantation in the central venous system of a hemodynamic wireless sensor for heart failure monitoring. Since all previous hemodynamic sensors were located in the pulmonary artery, there is no existing information about an optimal site in the central venous system.

**Methods:**

This study analysed data obtained from CT scans of most relevant anatomical features in the inferior vena cava. The most important parameters of the sites of interest were extracted, analysed statistically and compared, with the purpose to select an optimal site of implantation.

**Results:**

The results obtained show that the area comprised between the iliac bifurcation and the lower renal vein (and between the second and third lumbar veins) is the most suitable site of implantation for a hemodynamic sensor. Parameters such as its straight anatomy, diameter (21 mm) and link distance (106 mm) present it as a convenient location for implantation. Its procedure appears relatively easy, as access from the femoral vein is close to the site of interest. In addition, there are not major delicate structure in its surroundings that may pose a risk to the patient.

**Conclusion:**

This study concludes that the area between the iliac join and the lower renal vein (and the 2nd and 3rd lumbar veins) is an optimal site for the accommodation of a hemodynamic sensor.

## 1 Introduction

Among cardiovascular diseases, heart failure is a major cause of morbidity and mortality that affects at least 26 million people worldwide [1]. With an aging population, its prevalence is on the rise. Only in United States, 6.2 million people over 20 years old had heart failure between 2013 and 2016, in comparison to 5.7 million recorded between 2009 and 2012 [2]. It is also the first cause of hospitalization in the United States and Europe, and despite medical and technological advances in this area, there is a high rate of post-discharge death and reoccurrence after a first hospitalization [3].

Hospitalization due to heart failure represents a huge burden to the health system and is a key point for patients, as it is usually the start of a downfall in their health and quality of life [4].

Increase of ventricular filling pressure seems a reliable symptom of heart failure, arising weeks in advance of hospitalization [5]. In recent years, technological progress has provided implantable hemodynamic monitors that are able to track cardiac pressure in a continuous fashion. These devices have confirmed that reading cardiac pressure data can be used for optimization of management strategies to treat heart failure patients [6], [7], [8], [9].

Currently, all existing hemodynamic devices are implanted in the pulmonary artery. The reason for this is that pulmonary hypertension is a most common symptom in heart failure patients [10]. The main cause for pulmonary hypertension is the left heart disease (PHT WHO Classification – Group 2) with an increase in left heart filling pressures retrogradely leading to an increase in pulmonary pressures.

Despite this, pulmonary artery pressure is only linked to left ventricular pressure and left atrial pressure-mitral stenosis. It does not provide information on the right side of the heart and the complete systemic blood pressure. In addition, it is common in patients with heart failure to present lung diseases and thromboembolism, which can generate misreading of the actual heart pressure if measured at the pulmonary artery [11].

By contrast, pressure in the Central Venous System (CVS) presents a direct link to the heart’s right atrium. Reports have shown that pressure in the right part of the heart is an important guidance in heart failure patients [12], [13], [14]. It reflects the volume of blood returning to the heart and its ability to pump it back into the arterial system. Therefore, measurement of pressure in the CVS could predict a wider range of heart failure conditions.

This report is the first, to our knowledge, that studies the concept of CVS as a potential site of interest for implantation of a hemodynamic sensor. The potential to use the CVS as a collection point for pressure data could help compare similar data obtained from the pulmonary artery in patients with heart failure and extract new information on the condition and parameters to predict onsets of heart failure. In addition, Central Venous Pressure (CVP) could be linked to other conditions such as renal and liver failure.

As a first measure to consider implantation in the CVS, it is key to find an optimal site. Choosing the right anatomical feature where to place the implant will be dictated by several important factors. First, the sensor should be able to provide meaningful pressure readings that can be used for heart failure monitoring. Second, the sensor should be anchored firmly onto the chosen site, as rotation or migration could represent misreading of the sensor, or worse even, a threat to the patient’s health (migration to the lungs or, worse, to the heart, could cause serious complications in the patient). Finally, ease of implantation procedure and avoidance of delicate anatomical features would be desirable.

In order to gather anatomical data of the CVS, CT scans from patients diagnosed with peripheral vascular diseases were retrieved from the clinical archives at the University Hospital of Galway, Ireland. These CT scans present an ideal view of the CVS, in particular the Inferior Vena Cava (IVC), as most of the patients present complications in that area. Focusing in the IVC area suits the interest of this study, as it was preferred by clinical consensus to the Superior Vena Cava (SVC).

Clinical opinion favoured IVC as a site of implantation for several reasons. First, the SVC presents fewer potential locations suitable for implantation, due to its shorter length and connection to other vessels and anatomical structures. This does not happen in the IVC, where most of its length presents a straight feature that may lend itself with more ease to implantation. Second, the vena cava seems more fragile in the superior part than in the inferior part, and no previous devices have been implanted in this location. In fact, a procedure in this area involves potential damage to vessels that affect the brain directly, and the complications that this may arise are serious. By contrast, the IVC has proved to be an area of easy access for implantation of venous stents in the iliac zone, which creates a precedent. Finally, gravity and the direction of blood flow makes the SVC a difficult site of implantation for the clinician, as blood flow would attract the device towards the heart. Although both the SVC and IVC are connected to the right atrium of the heart, the former does it from above and the latter from below the heart. Blood in the vena cava flows in the direction of the heart, and therefore for the SVC it does it so in the direction of gravity, whereas for the IVC is the opposite.

Therefore, this article will show the most characteristic features of sites of interest in the IVC, which are feasible implantation sites. Analysis of their dimensions, anatomical features and potential vessel occlusion revealed the optimal selection of a site of implantation in the CVS.

## 2 Methods

20 CT scans from patients diagnosed with peripheral vascular disease were used to take measurements in the IVC area of the vena cava. Basic information on the patients whose scans were used is shown in Table 1. These CT scans were taken from the archive at the University Hospital Galway, Ireland.

**Table 1:** Basic information of patients whose CT scans were used for this study.

AGE	Gender	Pathology
66.5±10.17	95% M; 5% F	Peripheral Vascular Disease (n = 16); Abdominal Aortic Aneurysm (n = 4)

**Fig. 1:**
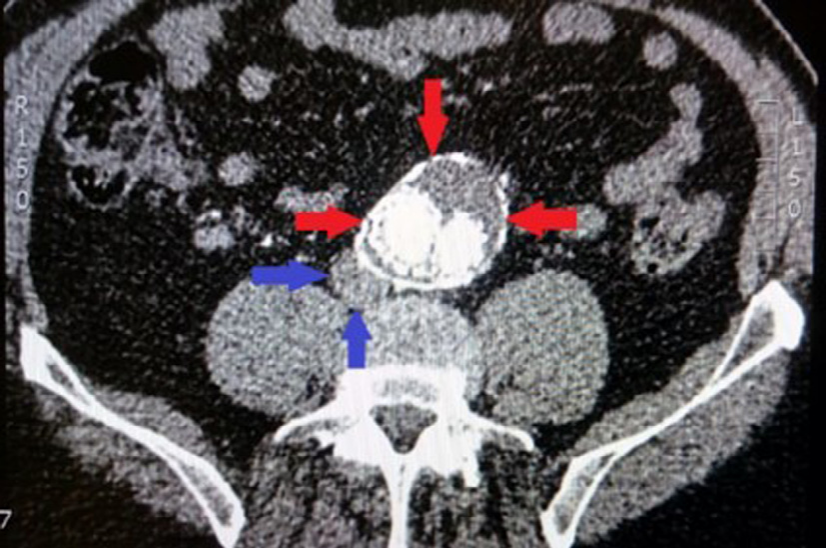
Image of a patient affected of an abdominal aortic aneurysm. The red arrows highlight the presence of the aneurysm, whereas the blue arrows point at the location of the IVC. The latter is compressed by the bulge of the aneurysm.

Of the 20 patients, four were discarded as they presented an abdominal aortic aneurysm. This condition does not allow proper visualization of the IVC, as the vein appeared compressed, as shown in Fig. 1, and therefore it was not possible to obtain accurate measurements of its diameter or length.

**Fig. 2:**
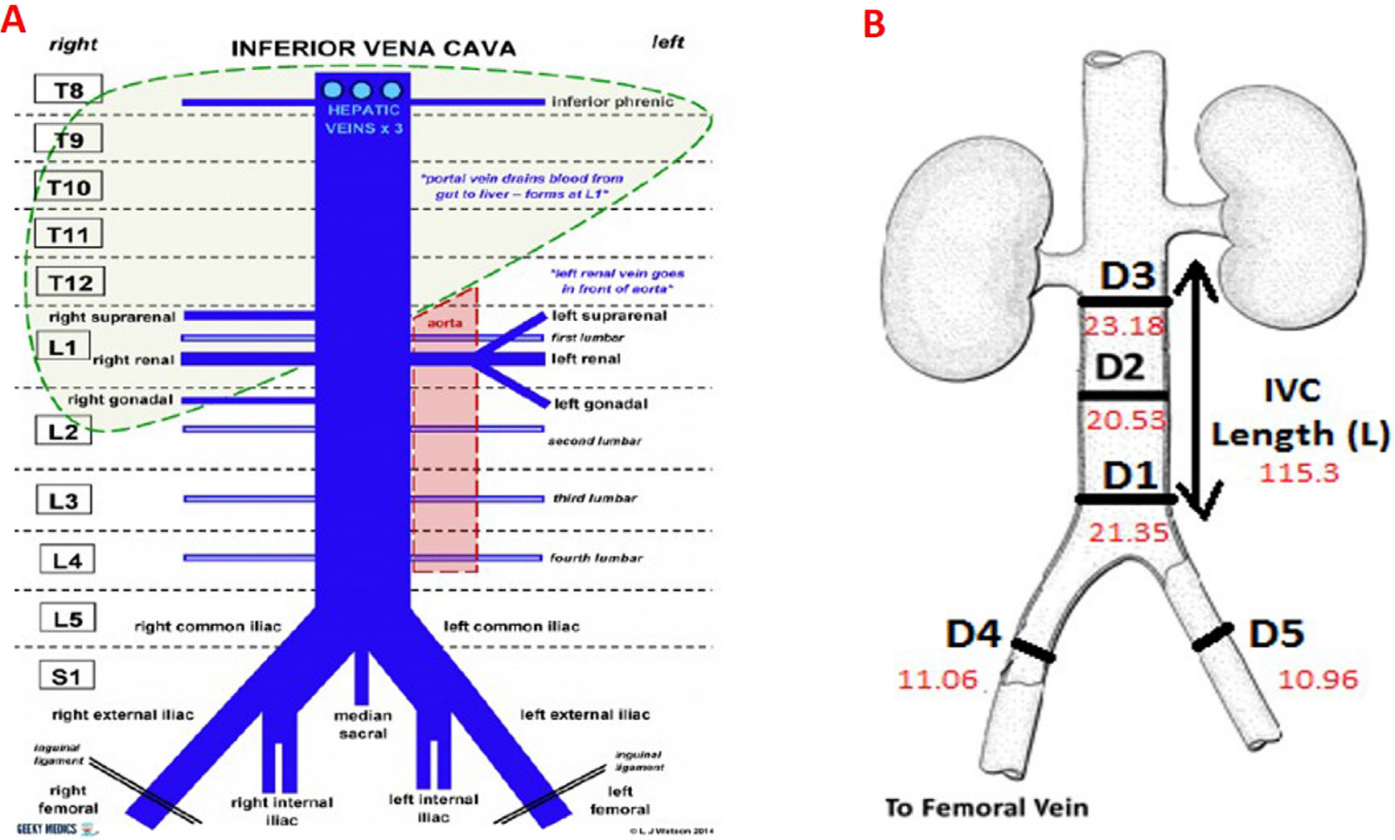
Inferior Vena Cava Tributaries: (A) Geeky Medics; Representation of the IVC with the most relevant features studied and their diameters in mm [15]. (B). D1, portion of the IVC immediately superior to the iliac bifurcation. D2, a midway location in the IVC between the lower renal vein and the iliac bifurcation D3, the site of the IVC immediately inferior to the right renal vein. D4 (right) and D5 (left) external iliac veins.

As for the IVC data obtained, measurements focused in the following areas of interest, shown also in Fig. 2.

**Fig. 3:**
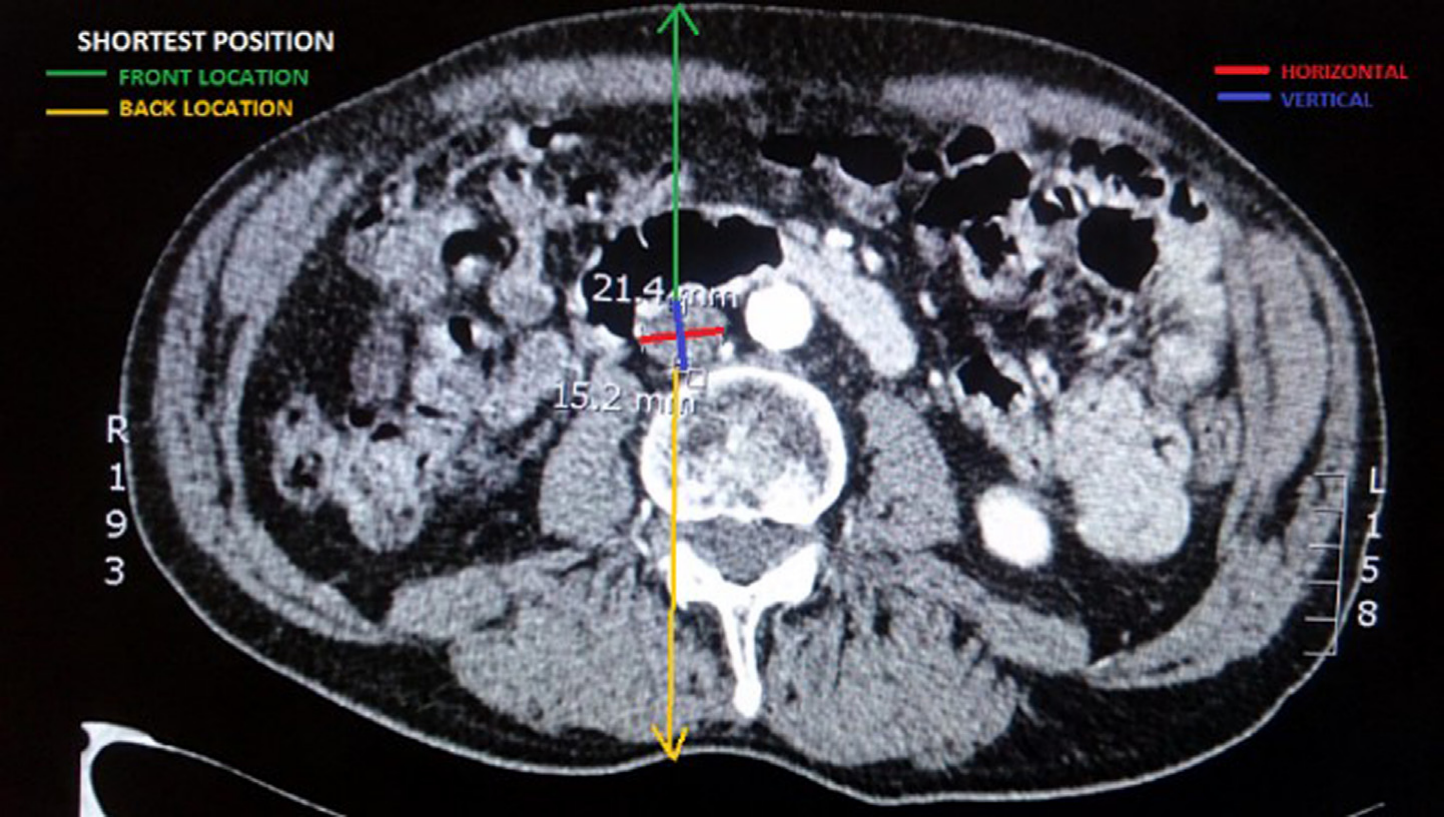
CT scan showing how measurements of diameter and link distances were taken. Link distance was measured both to the front and the back of the patient, as wireless collection of data can be done from either side of the patient.

The main parameters extracted from the CT images were two: diameter and link distance. Link distance is defined as the distance of the site of interest to the surface of the skin of the patient. An optimal link distance is important from the point of view of wireless data collection from the implant. Fig. 3 shows how measurements of diameter and link distances were performed. Measurements of diameters were done both horizontally and vertically. As for link distances, they were measured both from the back and the front of the patient to the most outward point of the vessel.

In addition to these measurements, calculation of potential vessel occlusion (VO) was carried out. This was done by considering the veins to have a circular cross-sectional area. Then, taking as a reference a haemodynamic sensor with a cross section of 8 mm^2^, the vessel occlusion was calculated as the proportion between the sensor cross sectional area and that of the vessel:

$$\mathrm{VO}=\frac{A_{\mathrm{sensor}}}{A_{\mathrm{vessel}}}\%$$

**Fig. 4:**
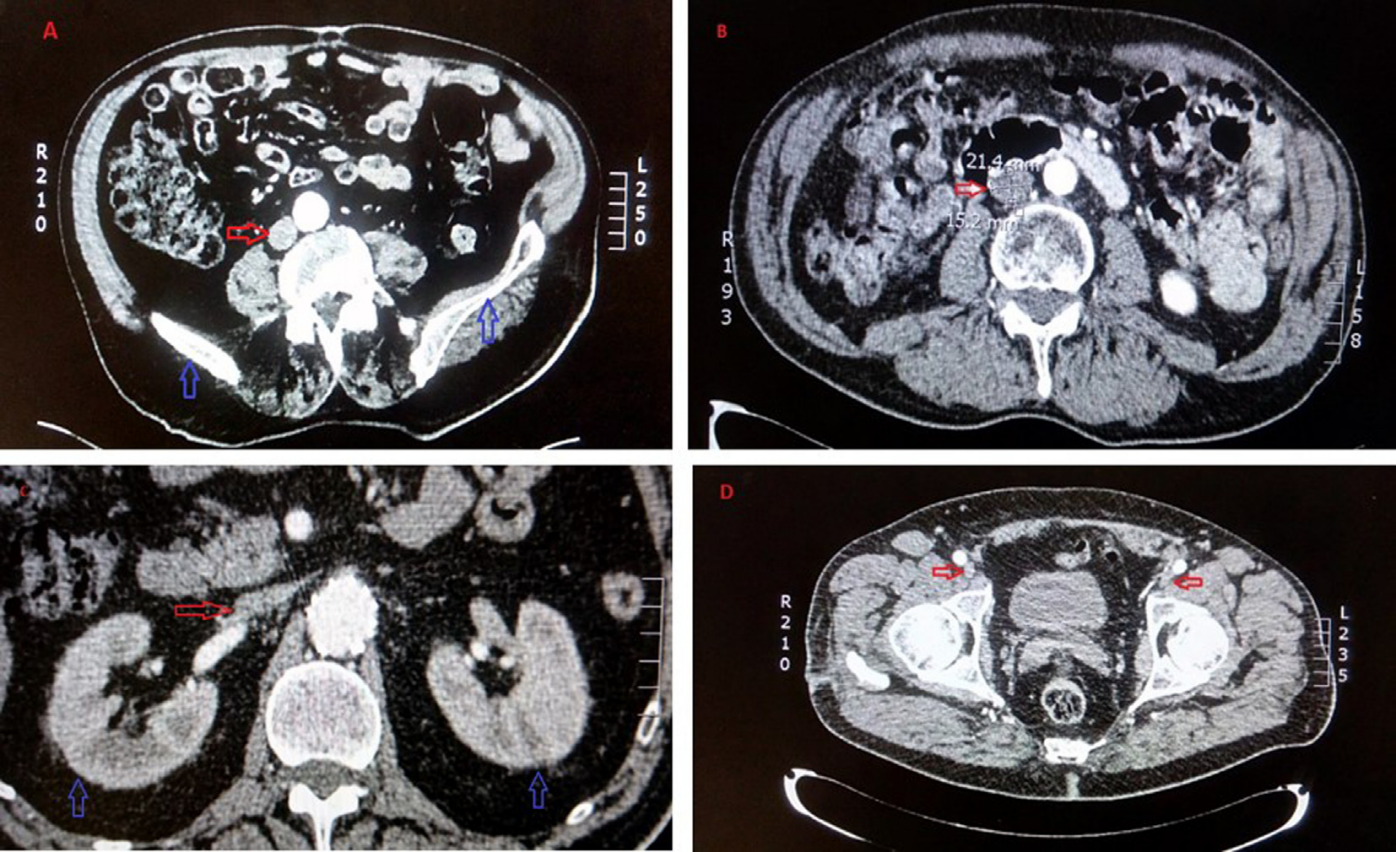
CT Scans of a patient with peripheral vascular disease. This image represents snapshots of all diameter locations considered: Fig. 4 (A) illustrates D1 location (diameter immediately superior to iliac bifurcation). It can be visualized in parallel to the aorta in contrast. The pelvis in contrast (indicated in blue arrows) is the landmark for D1 location. B illustrates D2 location (midway between the lower renal vein and iliac bifurcation). Further inside the axial view, the kidneys can be visualized. D2 area is surrounded by one-two pairs of lumbar veins. These veins cannot be visualized in the CT scans. Hence, the D2 location is found further inside D1 before the kidneys come into picture. C represents D3 location (diameter immediately inferior to the right renal vein). The red arrow indicates IVC just before it joins the right renal vein to the kidney. The blue arrows point to the location of kidneys. D shows left and right femoral veins indicated in red arrows. Above these arrows, the two bright spots show the aorta in contrast. These two femoral veins (indicated by red arrows) and bright spots further join inside to become IVC and aorta respectively.

Fig. 4 below illustrates all diameter locations (D1 to D5) in patients with peripheral vascular disease measured on the CT scans using a software Impax Client.

### 2.1 Sequence of measurement

In this axial view of CT, the first two locations spotted were D4 and D5 (femoral veins joining to the right and left external iliac veins). Further inside the axial view, the pelvis appears in contrast, which gives an indication of D1 location. Above this location, the IVC changes its shape (oval) as it needs to accommodate the renal inflow by joining the right renal vein. The D2 is spotted before the IVC changes its shape and joins the kidneys. A few more steps inside the axial view, D3 location can be measured before the IVC connects to the kidney. The supra-renal IVC locations were not considered as expert clinicians recommended “devices below the kidney” are easy to implant and avoids potential risks to the kidney (renal) and liver (hepatic) functions.

## 3 Results

In order to find the optimal site of implantation within the CVS, several factors were taken in consideration. These are: anatomical features of the vena cava, to find an optimal geometry for good sensor anchoring; diameter of the vessel, to ensure no obstruction of blood flow and proper design of the anchor; link distance, for clear wireless readings. As mentioned in the previous section, five locations were considered for discussion as potential sites of implantation, and the measurements obtained from the CT scans focused around those key locations (see Fig. 2B for locations D1, D2, D3, D4 and D5). From these measurements, several parameters were obtained: vessel diameter, cross sectional area, vessel occlusion and link distance. The following sections will detail the values found.

### 3.1 Size of veins

**Fig. 5:**
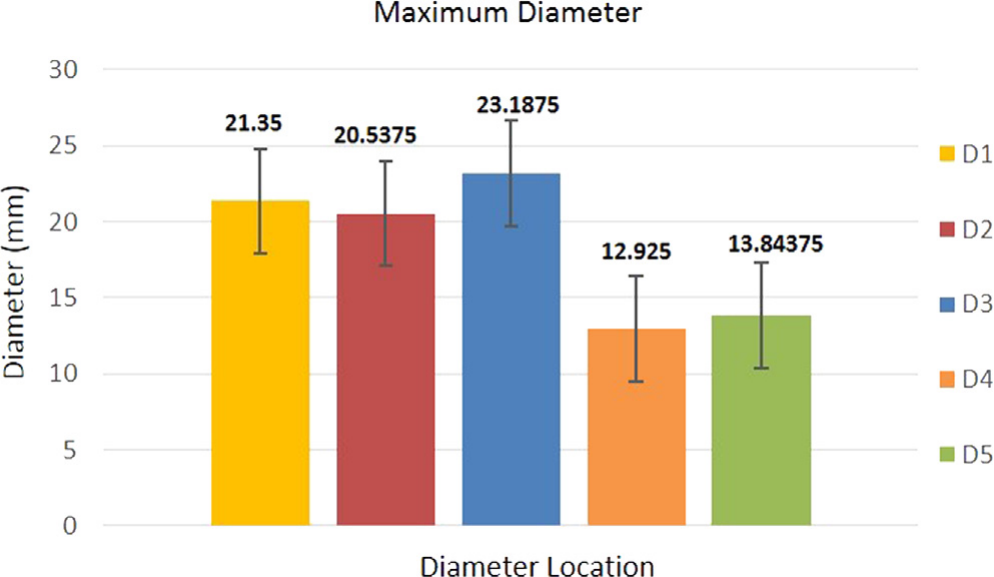
Maximum Mean Diameter of IVC (n = 16).

The diameters plotted for 16 patients are illustrated in Fig. 5 and summarized in Table 2. As it was expected D4 and D5, corresponding to the diameters of the external iliac veins, present the smallest values. At the other opposite range of values, D3 shows the maximum diameter measured. This was also expected, as it is known that the IVC has a straight anatomy in the portion between the iliac bifurcation and the renal veins, but that it normally expands slightly in diameter as it reaches the kidneys area.

**Table 2:** Diameters of IVC with one standard deviation, their respective range and cross-sectional area.

Location	Diameter (mm)^a^	Diameter Range (mm)	Cross sectional area^b^ (mm)^2^
D1	21 ± 3.5	18–25	268
D2	21 ± 2.9	17–23	245
D3	23 ± 3.2	20–26	283
D4	13 ± 2.3	10–15	113
D5	14 ± 1.9	12 – 15	120

a Data are presented as mean ± standard deviation. The population presented a normal distribution (n = 16).

b Assuming a vein of circular profile.

**Fig. 6:**
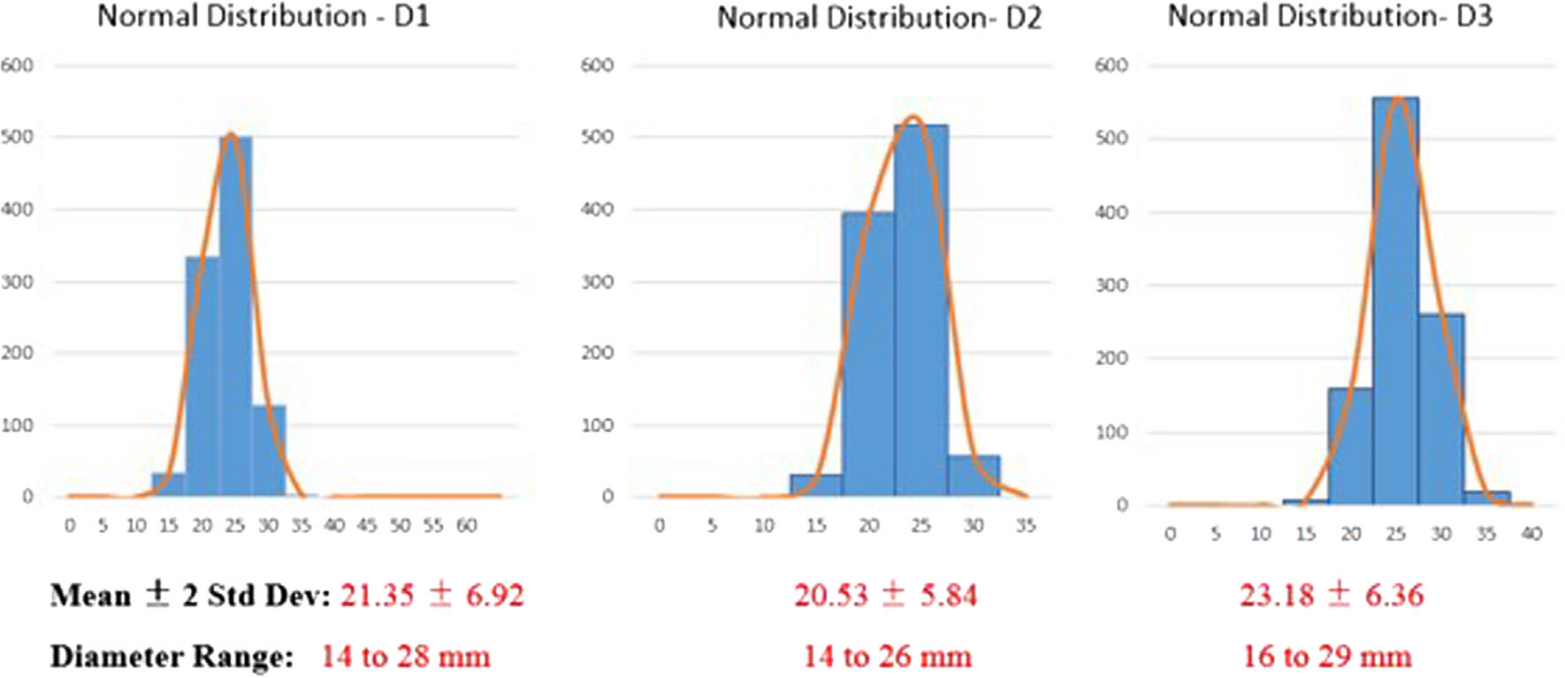
Normal Distribution plotted for diameters D1, D2 and D3.

Fig. 6 represents the study population i.e., IVC diameters (D1, D2, D3) of patients with peripheral vascular disease only (n = 16).

### 3.2 Vessel occlusion

A very unfavourable positioning of the hemodynamic sensor inside the vessel could cause major damage, producing occlusion and disturbing the blood flow. This condition needs to be avoided at all costs. It is possible to predict and prevent this threat by calculating the cross-sectional area of the vessels observed (table II) and selecting the ones that are wider than a minimum threshold (by industry standards in hemodynamic sensors, a 7% of the total blood flow in the vessel).

**Fig. 7:**
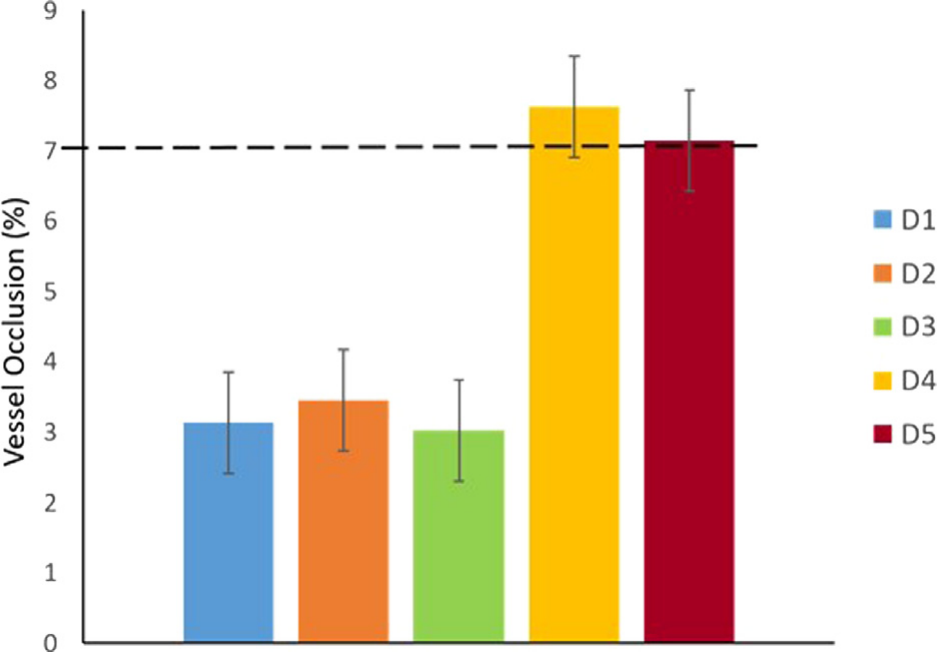
Vessel occlusion found for locations D1 to D5. Measured area of IVC at the sites, and calculation of occlusion produced by a sensor that is 8 mm^2^. A threshold of 7% marks the maximum level of occlusion allowed.

Fig. 7 shows the percentage vessel occlusion that a sensor with 8 mm^2^ footprint would cause. These values were obtained by calculating the cross-sectional area of the vessels at D1 to D5 (see material and methods section for further detail, eq. 1). The figure shows how of the five sites studied, D1 to D3 remain well within the safe limit, and no occlusion is likely to happen. This is not the case for D4 and D5: their cross-sectional area is indeed very small, which translates into a percentage of potential vessel occlusion (7.6 and 7.2 respectively) that could compromise the patient’s blood flow in that region.

### 3.3 Link distance

The link distance value is an important parameter, as it affects the quality of the readings that are collected with an external data unit. This unit needs to be placed in close proximity to the patient in order to avoid electrical interferences. Therefore, a short link distance would be beneficial. Table 3 shows the link distances obtained for all the locations considered as potential sites of implantation. In addition, link distances were measured both from the back and front of the patient, in order to establish which distance was the shortest in each location.

**Table 3:** Link distance values.

Location	Link Distance (mm)^a^^,^^b^	No. patients with shortest LD at front	No. of patients with shortest LD at back
D1	105 ± 24	8	8
D2	106 ± 26	8	8
D3	113 ± 23	8	8
D4	44 ± 19	16	0
D5	43 ± 18	16	0

a Population n = 16.

b Data presented as mean ± standard deviation.

Similarly, as to what was observed with diameters, D4 and D5 show the smallest link distances, whereas D3 again shows the highest value (Table 3). D1 and D2 link distances are almost identical and located in the middle range. Concerning front and back link distances, when comparing which is the shortest, D1, D2 and D3 had an equal number of patients with shortest distance back and front. This probably signifies easy access from either side. Interestingly, D4 and D5 showed both that their shortest link distance was at the front of the body.

In any of the cases, in patients with heart failure, where measurements of their hemodynamic parameters could take around twenty seconds each, it is preferred, due to comfort and stability of measurements, to have the reader located at the front side of the body, since this allows the patient to lie down while the reading takes place.

## 4 Discussion

Table 4 represents location considerations for an implant in the IVC. It presents a comparison between various diameter locations in terms of size/diameter, distance from the skin surface (link distance), number of branches surrounding the location and their respective positions from the heart.

**Table 4:** Location considerations in the Inferior Vena Cava.

**Infra-renal IVC Location**	**Size of the vessel/ Diameter ± Standard Deviation**	**Link Distance (mm)**	**Number of branches**	**Position from the heart**
D1: Immediately superior to iliac bifurcation	21.35 ± 3.46	105.3	2 (Right & Left external iliac veins)	Extremely Distal
D2: Midway between renal veins and iliac bifurcation	20.53 ± 2.92	105.5	5 (1 Right Gonadal, 4 Lumbar)	Distal
D3: Immediately inferior to right renal vein	23.18 ± 3.18	112.6	3 (Suprarenal, Renal, Gonadal veins)	Distal
D4: Right femoral vein	12.92 ± 2.28	43.6	2 (Right internal iliac vein, Ilio-lumbar vein)	Extremely Distal
D5: Left femoral vein	13.84 ± 1.85	42.8	2 (Left internal iliac vein, Ilio-lumbar vein)	Extremely Distal

According to the results shown in this report, D4 and D5, the two locations placed in the external iliac branches, are too narrow to hold an implantable sensor safely. The presence of an implant here would most likely compromise the blood flow. In addition, these areas sit in a body region (the pelvis) that is subject to considerable movement, which could compromise the anchoring of the sensor, or even perforate the wall of the vessel.

By contrast, D1, D2 and D3, which are located in the straightest anatomical region of the IVC, show generous cross-sectional areas that would avoid any potential occlusion by the presence of a hemodynamic sensor.

Their link distance is similar for D1 and D2, set at 105 mm, whereas D3 seems further from the skin surface, at 113 mm, but this difference may not be of enough significance to discard D3. In addition, all these three locations presented the possibility to place the wireless reader either at the back or at the front, as link distances in patients showed to be similar from either side. This could be of help to patients with additional impairments or mobility restrictions, as having different locations to place the external reader could be beneficial. Nevertheless, the preferred standard way to record pressure data is from the front, as it is easier to place a reader below the stomach area of the patient as the lie down or sit.

One important disadvantage to take into consideration about the D3 location is the proximity to the lower renal vein. Would the sensor migrate, it could potentially block the gonadal vein, or even the renal vein flow. This would be a high-risk possibility and should be avoided. In addition, the presence of the sensor could also provoke a dead zone in the blood flow to the renal vein. It is then preferable to discard them and consider either D1 or D2 as target areas of implantation.

Of the two remaining possible locations, D1 and D2, the latter seems a better option. D1 is closer to the iliac bifurcation and may cause some disruption in the blood flow from the iliac veins. Its position is also close to the pelvic joint, which, as in the case of D4 and D5, may subject any implant in this area to movement that could dislodge it. Another risk involved with the location D1 is its potential for compression due to the overlying iliac artery and underlying vertebral column.

The geometry of the site seems also less favourable than D2 to receive an implant. This is because the diameter of the IVC is tapered outwards in the direction of the kidneys, which may assist in the dislodgement of the sensor towards a wider lumen. In addition, the vein in D1 presents a curvature to join the femoral veins. D2, by contrast, lays in a very straight portion of the IVC, which may help in the design of a suitable anchor for the sensor to be implanted. These issues presented by the D1 location are not new, as they have been noticed in the implantation and use of venous stents in the iliac joint [16].

According to the results shown in this report, location D2, sitting between the iliac bifurcation and the lower renal vein (and between the second and third lumbar veins) is an optimal site for implantation of a hemodynamic sensor. This part of the IVC presents a straight profile, which could facilitate the design of an anchor fitting this anatomy. There are multitudes of stent designs that are based in circular shapes, and this may be a starting point for optimisation of the anchor design. In addition, access to D2 is easy, via the right femoral vein, and the distance to the entry point is short. Not only that, but there are no major delicate structures around the area that would suffer during the procedure.

The link distance from D2 to the front of the body (106 mm) fell in the middle range of all the measurements obtained, and it is considered optimal for a wireless reader. Finally, the diameter of this area is wide enough to admit comfortably a sensor without disruption of the blood flow, as the vessel occlusion value that was obtained showed (3.5% of the total size of the vein). Moreover, it presents a straightforward implantation procedure which is attractive to many physicians. Physicians have a vast experience of device implantation, particularly the IVC filters in the D2 area. Another advantage it has over D1 and D3 is the lower risk of blocking surrounding structures, i.e., presence of second and third pairs of lumbar veins that branch laterally and antero-posteriorly. Blocking these vessels is not as much a concern, as many lumbar veins branch laterally in the infra-renal portion of the vena cava and these will compensate for the blood flow if one or two of these vessels are blocked by the sensor.

### 4.1 Limitations

This study is limited by its retrospective nature and relatively small sample size. However, it presents a robust method of analysis for selection of an optimal site for implantation of the wireless sensor, the outcome of which was approved by clinicians at the University Hospital. All the CT scans were obtained from the archive at University College Hospital Galway. This study could have incorporated IVC measurements from a large patient population with heart failure, chronic obstructive pulmonary disease (COPD) or other related comorbidities. The IVC diameter is larger (distended) in patients with chronic heart failure and it does not collapse 100% during the respiratory cycle [14]. Unfortunately, there was no data in the archive that would shed light upon this or compare changes in the IVC diameter in patients with and without heart failure, PVD and AAA. One important thing to note in this study is that the diameters of IVC measured in patients with PVD are considered to be in the normal range. This means that IVC diameters in PVD patients do not reflect false measurements. Provided that this study included data of patients only with HF, we would not be able to conclude an optimal site for the placement of the central venous sensor as the IVC dynamics would vary in these patients. The design modification of the anchor with a higher outward radial force would compensate for this limitation in future. However, these factors cannot deny the fact that the study requires further clinical validation.

## 5 Conclusions

Hemodynamic sensors have proven, in the recent years, useful in the continuous monitoring of heart failure patients. Nevertheless, current implantation in the pulmonary artery may not provide complete information as it is only connected to the left side of the heart. By contrast, the central venous system may provide a wider set of data as its pressure is linked to both the right and left side of the heart.

The aim of this work was to assess the central venous system for suitable locations of a hemodynamic implant. In particular, it focused on the inferior vena cava as it presents several strong candidate locations that offer easy surgical access.

Study of the anatomical features of the inferior vena cava showed that the middle area comprised between the lower renal vein and the iliac join seems an ideal candidate for implantation of a hemodynamic sensor. The measurements obtained show that the diameter of this area is wide enough to accept a sensor without obstruction of blood flow. In addition, the link distance from this location to the surface of the skin seems ideal for the use of wireless recording of the implant.

These findings will be used for the design of a novel anchor suitable for deployment of a wireless sensor in the chosen IVC region. This design will be validated in acute and chronical studies with animals and compare with the standard implantation site in the pulmonary artery.

## CRediT authorship contribution statement

**Tejaswini Manavi:** Data curation, Formal analysis, Investigation, Methodology, Validation, Visualization, Writing - original draft. **Patricia Vazquez:** Investigation, Methodology, Validation, Visualization, Writing - original draft. **Muhammad Tubassam:** Data curation, Resources, Visualization. **Junaid Zafar:** Formal analysis, Investigation, Methodology, Validation, Visualization, Writing - review & editing. **Faisal Sharif:** Funding acquisition, Investigation, Resources, Validation, Writing - review & editing. **Haroon Zafar:** Conceptualization, Data curation, Formal analysis, Funding acquisition, Investigation, Methodology, Project administration, Supervision, Validation, Visualization, Writing - original draft.

## Declaration of Competing Interest

The authors declared that there is no conflict of interest.
